# A New Xanthone Glycoside from the Endolichenic Fungus *Sporormiella irregularis*

**DOI:** 10.3390/molecules21060764

**Published:** 2016-06-11

**Authors:** Bin-Jie Yang, Guo-Dong Chen, Yan-Jun Li, Dan Hu, Liang-Dong Guo, Ping Xiong, Hao Gao

**Affiliations:** 1 Department of Pharmaceutical Engineering, College of Materials and Energy, South China Agricultural University, Guangzhou 510640, China; 15920520642@163.com; 2 Institute of Traditional Chinese Medicine & Natural Products, College of Pharmacy, Jinan University, Guangzhou 510632, China; Liyanjun1014@163.com (Y.-J.L.); thudan@jnu.edu.cn (D.H.); tghao@jnu.edu.cn (H.G.); 3 State Key Laboratory of Mycology, Institute of Microbiology, Chinese Academy of Sciences, Beijing 100190, China; guold@sun.im.ac.cn

**Keywords:** xanthone glycoside, *Sporormiella irregularis*, endolichenic fungus, biosynthetic pathway

## Abstract

A new xanthone glycoside, sporormielloside (**1**), was isolated from an EtOAc extract of an endolichenic fungal strain *Sporormiella irregularis* (No. 71-11-4-1), along with two known xanthones (**2**, **3**). Their structures were determined by detailed spectroscopic analysis (IR, MS, and 1D- and 2D-NMR), a chemical method, and a comparison of NMR data with closely related compounds previously reported. According to the structures of isolated compounds, their plausible biosynthetic pathway was deduced.

## 1. Introduction

*Sporormiella* is a genus belonging to the family of Sporormiaceae with more than 80 species distributed across boreal and temperate regions of the world [1,2,3,4]. Some species of the genus are saprobes, and others are endophytic in living plants, fungi, and lichens [5,6,7,8]. Previous chemical investigation of this genus led to isolation of polyketides (such as xanthones (sporormiellins A–C, sporormiellones A, B, microsphaeropsone A, brocaenol B) [5,9], chromones (corymbiferone C, corymbiferan lactone E, corymbiferone) [5], macrocyclic lactone (sporostatin) [10], organic acids (including zaragozic acid B, L731-127, L731-128, sporovexins A, B, and sporminarins A, B) [11,12,13]), triterpenoids (including FR171456, FR173945 [14,15], and (2a*R*,2a^1^*R*,3*S*,5a*R*,6b*S*,9*S*,10a*R*,12b*S*)-9-hydroxy-2a^1^,3,6b,10,10,12b-hexamethyl-1,2,2a,2a^1^,3,5a,6,6b,7,8,9,10,10a,11,12,12b-hexadecahydro-4*H*-cyclopenta[*de*]naphtho[2,1-*g*]chromen-4-one [16]), steroids (including 22*E*-3,7-epoxy-5,10:8,9-disecoergosta-9(10),22-diene-5,8-dione, 22*E*-5α,6α-epoxyergosta-8(14),22-diene-3β,7α-diol, 22*E*-5α,6α-epoxyergosta-8(9),22-diene-3β,7α-diol, 22*E*-7*α*-methoxy-5*α*,6*α*-epoxyergosta-8(14),22-dien-3β-ol, 22*E*-3β-hydroxy-5α,6α-epoxyergosta-22-en-7-one, 22*E*-ergosta-7,22-diene-3β,5α,6β-triol, 22*E*-6β-methoxyergosta-7,22-diene-3β,5α-diol, 22*E*-5α,8α-epidioxyergosta-6,22-dien-3β-ol and 22*E*-ergosta-4,6,8(14),22-tetraen-3-one [17]), and the nitrogenous compounds (including terezines A–D [18], similin B [19], sporovexin C [12], and (2*Z*,4*E*,6*E*)-3-hydroxy-*N*-(1,11a,11b-trihydroxy-3,6,9-trioxodecahydro-1*H*-pyrazino[1,2-*a*]pyrrolo[2,1-*c*]pyrazin-10-yl)octadeca-2,4,6-trienamide [20]).

In our previous chemical investigation of this genus *Sporormiella*, a series of xanthones (sporormiellins A–C, sporormiellones A, B, and microsphaeropsone A) were isolated from *S. minima* (No. 66-3-4-2) [9]. Notably, sporormiellin A is the first discovered tetrahydrofuran-fused furochromone with an unprecedented tetracyclic skeleton. As a part of our continuing search for more xanthones, a chemical investigation of another species (*S. irregularis* (No. 71-11-4-1)) from this genus was carried out, which led to isolation of a new xanthone glycoside, sporormielloside (**1**), along with two known biogenetically related compounds, calyxanthone-8-methyl ether (**2**) and endocrocin (**3**). Compound **2** is a known compound; however, there is no reference reporting its NMR data. Therefore, the details of the isolation and structural elucidation of these isolated compounds are reported herein.

## 2. Results and Discussion

**Figure 1:**
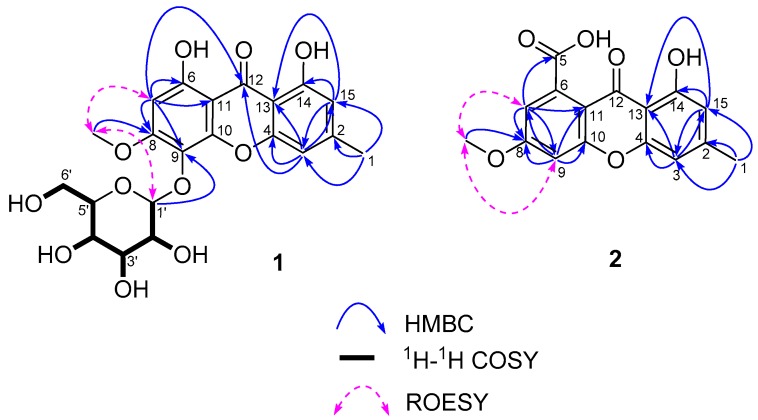
Key ^1^H-^1^H COSY, HMBC and ROESY correlations of **1** and **2**.

**Figure 2:**
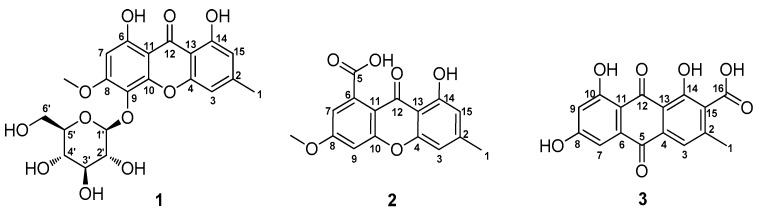
Chemical structures of **1**–**3.**

Compound **1** was obtained as a yellow amorphous powder. The quasi-molecular ion at *m/z* 451.1240 [M + H]^+^ by HRESIMS indicated the molecular formula of **1** was C_21_H_22_O_11_ with 11 degrees of unsaturation. The ^1^H-NMR spectrum (DMSO-*d*_6_, Figure S1) displayed six exchangeable protons (δ_H_ 11.76 (1H, br s), 11.64 (1H, br s), 5.14 (1H, d, *J* = 4.9 Hz, 2′-OH), 5.01 (1H, d, *J* = 4.8 Hz, 3′-OH), 4.92 (1H, d, *J* = 5.1 Hz, 4′-OH), and 4.26 (1H, t, *J* = 5.6 Hz, 6′-OH)), three aromatic protons (δ_H_ 6.92 (1H, br s, H-3), 6.65 (1H, br s, H-15), and 6.58 (1H, s, H-7)), five sp^3^ methine protons (including one anomeric proton δ_H_ 4.84 (1H, d, *J* = 7.6 Hz, H-1′)), one sp^3^ methylene group (δ_H_ 3.60 (1H, ddd, *J* = 11.6, 5.6, 1.8 Hz, H-6′a) and 3.39 (1H, ddd, *J* = 11.6, 5.8, 5.6 Hz, H-6′b)), and two methyl groups (δ_H_ 3.92 (3H, s, 8-OCH_3_), 2.39 (3H, br s, H-1)). The ^13^C-NMR and DEPT-135 spectra (Figure S2) of **1** showed 21 carbon signals including a ketone carbonyl (δ_C_ 183.6), twelve aromatic carbons (δ_C_ 160.1, 159.9, 157.6, 155.5, 149.3, 148.5, 125.1, 111.2, 107.9, 104.7, 101.3, and 95.5), two methyl carbons (including one oxygenated (δ_C_ 56.7)), and a set of hexose moiety carbons (δ_C_ 103.6, 77.3, 76.5, 74.2, 69.9, and 61.1). According to the analysis of coupling constants (^3^*J*_H-2′,H-3′_ = 8.6 Hz, ^3^*J*_H-3′,H-4′_ = 8.6 Hz, and ^3^*J*_H-4′,H-5′_ = 9.6 Hz), the analysis of ^1^H-^1^H COSY (Figure 1), and a comparison with the previously reported [21,22] ^13^C-NMR data of the glucopyranoside unit, the hexose moiety of **1** was identified as glucopyranoside (Glu). After acid hydrolysis and derivatization of **1**, the HPLC analysis revealed the presence of d-glucoses and compared them with derivatives obtained by the same method with standard monosaccharides [23]. The Glu unit in **1** was attached to the aglycone via a β-linkage on the basis of the coupling constant of the anomeric proton located at δ_H_ 4.84 (1H, d, *J* = 7.6 Hz, H-1′). The connection position of Glu unit to aglycone was established at C-9 on the basis of the HMBC correlation from H-1′ to C-9. Combined with the molecular formula and chemical shifts of ^1^H and ^13^C, the structure of aglycone was established based on the key HMBC correlations from H-1 to C-2/C-3/C-15, from H-3 to C-4/C-12/C-13/C-15, from H-15 to C-3/C-13/C-14, from H-7 to C-6/C-8/C-9/C-11/C-12, from 8-OCH_3_ to C-8, and the key ROESY correlations between 8-OCH_3_ and H-7/H-1′ (as shown in Figure 1). Therefore, the structure of **1** was elucidated as shown in Figure 2, which was a new xanthone glycoside called sporormielloside. The assignments of all proton and carbon resonances are provided in Table 1.

Compound **2** was obtained as a yellowish amorphous powder. The quasi-molecular ion at *m/z* 323.0527 [M + Na]^+^ by HRESIMS indicated the molecular formula of **2** was C_16_H_12_O_6_ with 11 degrees of unsaturation. The ^1^H-NMR spectrum (DMSO-*d*_6_, Figure S7) displayed two exchangeable protons (δ_H_ 13.23 (1H, br s), 12.33 (1H, brs)), four aromatic protons (δ_H_ 7.20 (1H, d, *J* = 2.4 Hz, H-9), 6.99 (1H, d, *J* = 2.4 Hz, H-7), 6.88 (1H, br s, H-3), and 6.67 (1H, br s, H-15)), and two sp^3^ methyl groups (δ_H_ 3.96 (3H, s, 8-OCH_3_), 2.41 (3H, s, H-1)). The ^13^C-NMR and DEPT-135 spectra (Figure S8) showed 16 signals, assigned two carbonyl carbons (a ketone one (δ_C_ 179.2) and a carboxylic acid/ester one (δ_C_ 168.8)), twelve sp^2^ aromatic carbons, and two methyl carbons (including an oxygenated one (δ_C_ 56.6)). Combined with the molecular formula and chemical shifts of ^1^H and ^13^C, the key HMBC correlations from H-1 to C-2/C-3/C-15, from H-3 to C-4/C-13/C-15, from H-15 to C-3/C-13/C-14, from H-7 to C-5/C-8/C-9/C-11, from H-9 to C-7/C-8/C-10/C-11, from 8-OCH_3_ to C-8, and the key ROESY correlations between 8-OCH_3_ and H-7/H-9 (as shown in Figure 1) established the planar structure of **2**, which was a methyl esterified derivative of calyxanthone [24] at C-8. Therefore, the structure of **2** was elucidated as the same as that of the known compound (calyxanthone-8-methyl ether). The assignments of NMR data of **2** are provided in Table 2.

Compound **3** was obtained as a yellow amorphous powder. The quasi-molecular ion at *m/z* 313.0345 [M − H]^−^ by HRESIMS indicated the molecular formula of **3** was C_16_H_10_O_7_ with 12 degrees of unsaturation. The ^1^H-NMR and ^13^C-NMR spectrum (DMSO-*d*_6_) are the same as that of endocrocin [25].

**Scheme 1:**
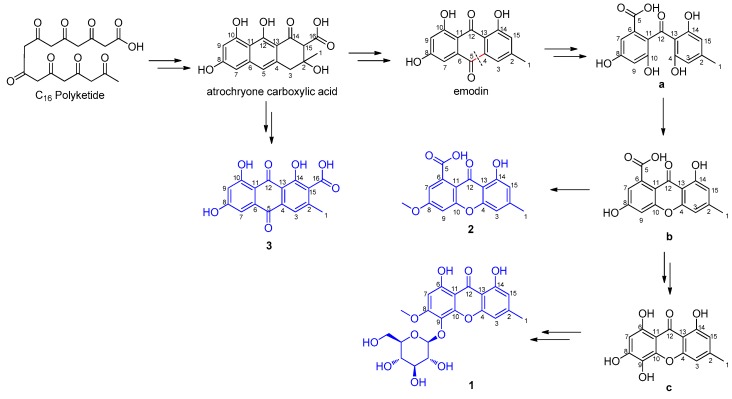
The plausible biosynthetic pathway of **1**–**3**.

Based on the structural features of compounds **1**–**3**, the plausible biosynthetic pathway of them was deduced (Scheme 1). The C_16_-octaketide produced by non-reducing polyketide synthase undergoes cyclization to yield atrochryone carboxylic acid [26], which is either autoxidized to endocrocin (**3**) or undergoes dehydration first followed by decarboxylation and spontaneous oxidation to give emodin [27,28]. The resulting emodin will be enzymatically transformed into intermediate **a** by oxidative ring opening between C-4 and C-5 [26,29], and subsequent dehydration will give intermediate **b**. Compound **2** may be derived from intermediate **b** via methylation. After decarboxylation and oxidation, intermediate **b** may transform into intermediate **c**, and **1** may be derived from intermediate **c** via methylation and glycosylation [26].

## 3. Materials and Methods

### 3.1. Chemicals

l-cysteine methyl ester hydrochloride, *o*-tolyl isothiocyanate, d-glucose (d-Glc), l-glucose (l-Glc), and DMSO-*d*_6_ were purchased from Sigma-Aldrich Chemical Co. Ltd. (Saint Louis, MO, USA). Methanol (MeOH) was purchased from Yuwang Industrial Co. Ltd. (Yucheng, China). Formic acid (HCOOH) was obtained from Kemiou Chemical Reagent Co. Ltd. (Tianjin, China).

### 3.2. General Experimental Procedures

UV data were recorded using a JASCO V-550 UV/vis spectrometer (Jasco International Co. Ltd., Tokyo, Japan). IR data were recorded on a JASCO FT/IR-480 plus spectrometer (Jasco International Co. Ltd.). Optical rotations were measured on a JASCO P1020 digital polarimeter (Jasco International Co. Ltd.). The ESIMS spectra were performed on a Bruker amaZon SL mass spectrometer (Bruker Daltonics Int., Boston, MA, USA), and the HRESIMS spectra were obtained on a Waters Synapt G2 mass spectrometer (Waters Corporation, Milford, MA, USA). 1D and 2D NMR spectra were acquired with Bruker AV 400 spectrometers (Bruker BioSpin Group, Faellanden, Switzerland) using the solvent signals (DMSO-*d*_6_: δ_H_ 2.50/δ_C_ 39.5) as internal standards. The analytical HPLC was performed on a Dionex HPLC system equipped with an Ultimate 3000 pump, an Ultimate 3000 DAD, an Ultimate 3000 Column Compartment, an Ultimate 3000 autosampler (Thermo Fisher Scientific Inc., Sunnyvale, CA, USA), and an Alltech (Grace) 2000ES evaporative light scattering detector (Alltech International Inc., Vienna, VA, USA) using a Phenomenex Gemini C_18_ column (4.6 mm × 250 mm, 5 μm) (Phenomenex Inc., Los Angeles, CA, USA). Semi-preparative HPLC was performed on a Dionex HPLC system, which was equipped with an Ultimate 3000 pump, and an Ultimate 3000 RS variable wavelength detector using a Phenomenex Gemini C_18_ column (10.0 mm × 250 mm, 5 μm) (Phenomenex Inc.). The medium pressure liquid chromatography (MPLC) was performed on ODS (60–80 μm, YMC Co. Ltd., Tokyo, Japan) and equipped with a dual pump gradient system, a UV preparative detector, and a Dr Flash II fraction collector system (Lisui E-Tech Co. Ltd., Shanghai, China). The organic solvent was evaporated with an EYELA rotary evaporator N-1100 system (Tokyo Rikakikai Co. Ltd., Shanghai, China).

### 3.3. Fungus Material

The strain numbered as 71-11-4-1 was isolated from the lichen *Usnea mutabilis Stirt*, which was collected in Zixishan Mountain, Yunnan province, China, in November 2006. The strain was identified as *S. irregularis* based on the morphological characters by one of our authors (L.-D.G.). The fungal strain was cultured on slants of potato dextrose agar (PDA) at 25 °C for 5 days. Agar plugs were used to inoculate nine Erlenmeyer flasks (250 mL), each containing 100 mL of potato dextrose broth (PDB). The nine flasks of the inoculated media were incubated at 25 °C on a rotary shaker at 200 rpm for five days to prepare the seed culture. Fermentation was carried out in 30 Erlenmeyer flasks (500 mL), each containing 70 g of rice. Distilled H_2_O (105 mL) was added to each flask, and the rice was soaked overnight before autoclaving at 121 °C for 30 min. After cooling to room temperature, each flask was inoculated with 10 mL of the spore inoculum and incubated at 25 °C for 40 days.

### 3.4. Extraction and Isolation

The culture was extracted four times with EtOAc, and the organic solvent was evaporated in a vacuum to afford the dry crude extract (29.05 g). The crude extract was dissolved in 90% *v*/*v* aqueous MeOH (500 mL) and partitioned against the same volume of cyclohexane to afford a cyclohexane fraction (C, 13.42 g) and an aqueous MeOH fraction (W, 12.38 g). The aqueous MeOH fraction (W, 12.38 g) was separated by MPLC eluting with MeOH–H_2_O (30:70, 50:50, 70:30, and 100:0, *v*/*v*) to afford four fractions (W1 to W4). Fraction W3 (753.4 mg) was further separated by MPLC with a gradient of MeOH–H_2_O to yield 9 subfractions (W3a–W3i) and **1** (11.7 mg). Fraction W2 (1.14 g) was further separated by MPLC with a gradient of MeOH–H_2_O to yield 19 subfractions (W2a to W2s). Subfraction W2m (50.0 mg) was purified by semi-preparative HPLC with MeOH–H_2_O–HCOOH (80:20:0.1, *v*/*v*) at a flow rate of 3 mL/min to yield **2** (4.0 mg). Subfraction W2i (66.1 mg) was purified with semi-preparative HPLC using MeOH–H_2_O–HCOOH (60:40:0.1, *v*/*v*) at flow rate of 3 mL/min to yield **3** (9.0 mg).

*Sporormielloside* (**1**) (Figure 2): Yellow amorphous powder; ${\left[\alpha \right]}_{\text{D}}^{25}$ = –46.3 (*c* = 0.08, CHCl_3_–MeOH = 1:2); UV (MeOH) λ_max_ (log *ε*) 206 (4.66), 232 (4.24), 256 (4.42), 336 (4.13) nm; IR (KBr) υ_max_ 3421, 2906, 2360, 2339, 1632, 1608, 1515, 1370, 1267, 1209, 1160, 1122, 1071, 1024, 552 cm^−1^; ESIMS (positive): *m/z* 473 [M + Na]^+^, 923 [2M + Na]^+^; HRESIMS (positive): *m/z* 451.1240 [M + H]^+^ (calcd. for C_21_H_23_O_11_, 451.1240); ^1^H- and ^13^C-NMR data see Table 1.

*Calyxanthone-8-methyl ether* (**2**) (Figure 2): Yellowish amorphous powder; UV (MeOH) λ_max_ (log ε) 202 (4.53), 236 (4.62), 251 (4.51), 302 (4.37) 351 (3.96) nm; IR (KBr) υ_max_ 3446, 3248, 2360, 2335, 1739, 1650, 1600, 1571, 1504, 1455, 1423, 1394, 1273, 1217, 1179, 1154, 1128, 1016, 829, 687 cm^−1^; ESIMS (positive): *m/z* 301 [M + H]^+^, 323 [M + Na]^+^; ESIMS (negative) *m/z* 299 [M − H]^−^, 255 [M ‒ COOH]^‒^; HRESIMS (positive): *m/z* 323.0527 [M + Na]^+^ (calcd. for C_16_H_12_O_6_Na, 323.0532); ^1^H- and ^13^C-NMR data see Table 2.

*Endocrocin* (**3**) (Figure 2): Yellow amorphous powder; UV (MeOH) λ_max_ (log ε) 203 (4.55), 226 (4.65), 375 (3.50), 440 (4.26) nm; IR (KBr) υ_max_ 3377, 2361, 2339, 1715, 1671, 1622, 1384, 1255, 1206, 1171, 756 cm^−1^; ESIMS (negative) *m/z* 313 [M − H]^−^; HRESIMS (negative): *m/z* 313.0345 [M − H]^−^ (calcd. for C_16_H_9_O_7_, 313.0348).

### 3.5. Acid Hydrolysis

Acid hydrolysis was performed according to the method described by Tanaka *et al.* with standard monosaccharides [23]. Compound **1** (1.0 mg) was hydrolyzed with 2 M of HCl for 1 h at 90 °C. After it was extracted with EtOAc twice, the H_2_O layer was evaporated *in vacuo* to furnish a monosaccharide residue using a rotary evaporator. The residue was dissolved in pyridine (1.0 mL) containing L-cysteine methyl ester hydrochloride (1.0 mg) and heated at 60 °C. After 1 h, 10 μL of *o*-tolyl isothiocyanate was added to the reaction mixture and further reacted at 60 °C for 1 h. Then, the reaction mixture was directly analyzed by the Dionex HPLC system and detected by an UV detector (at 254 nm). Analytical HPLC was performed on the Phenomenex Gemini C_18_ column with isocratic elution of CH_3_CN–H_2_O (40:60, *v*/*v*) for 40 min at a flow rate of 0.8 mL/min. The standard monosaccharides of D-Glc and L-Glc were subjected to the same method.

## 4. Conclusions

Xanthones are commonly found in higher plants, fungi, and lichens [30,31]. However, only eight xanthone aglycones have been isolated from *Sporormiella*. Xanthone glycosides are mainly isolated from plants such as Gentianaceae and Guttiferae [32,33] but are rarely reported from fungi, except for *Phomopsis* sp. ZH76 [34], *Paecilomyces cinnamomeus* BCC 9616 [35], and *Aschersonia coffeae* Henn. Bcc 28712 [36]. The isolation of sporormielloside (**1**), which is the first report for xanthone glycoside isolated from *Sporormiella*. Owing to a lack of any references to calyxanthone-8-methyl ether (**2**) in the literature, moreover, the assignments of NMR data of **2** are provided here for the first time.

## Figures and Tables

**Table 1:** The ^1^H-NMR (400 MHz) and ^13^C-NMR (100 MHz) data of compound **1** (in DMSO-*d*_6_).

No.	1				
	δ_C_, Mult	δ_H_, Mult, (*J* in Hz)	^1^H-^1^H COSY	HMBC	ROESY
1	21.9, CH_3_	2.39, br s	3, 15	2, 3, 15	
2	149.3, qC				
3	107.9, CH	6.92, br s	1, 15	1, 4, 12, 13, 15	
4	155.5, qC				
6	157.6, qC				
7	95.5, CH	6.58, s		6, 8, 9, 11, 12	8-OCH_3_
8	160.1, qC				
9	125.1, qC				
10	148.5, qC				
11	101.3 qC				
12	183.6, qC				
13	104.7, qC				
14	159.9, qC				
15	111.2, CH	6.65, br s	1, 3	1, 3, 13, 14	
8-OCH_3_	56.7, CH_3_	3.92, s		8	7, 1′
6-OH *		11.76, br s			
14-OH *		11.64, br s			
1′	103.6, CH	4.84, d, (7.6)	2′	9	3′, 5′, 8-OCH_3_
2′	74.2, CH	3.32, overlapped	1′, 3′, 2′-OH	1′, 3′, 4′	
3′	76.5, CH	3.24, td, (8.6, 4.8)	2′, 4′, 3′-OH	2′, 4′	1′, 5′
4′	69.9, CH	3.16, m	3′, 5′, 4′-OH	3′, 5′, 6′	
5′	77.3, CH	3.09, ddd, (9.6, 5.8, 1.8)	4′, 6′a, 6′b	1′, 4′	1′, 3′
6′	61.1, CH_2_	3.60, ddd, (11.6, 5.6, 1.8), a 3.39, ddd, (11.6, 5.8, 5.6), b	5′, 6′b, 6′-OH 5′, 6′a, 6′-OH		
2′-OH		5.14, d, (4.9)	2′		
3′-OH		5.01, d, (4.8)	3′		
4′-OH		4.92, d, (5.1)	4′		
6′-OH		4.26, t, (5.6)	6′a, 6′b		

*: The assignment maybe exchanged.

**Table 2:** The ^1^H-NMR (400 MHz) and ^13^C-NMR (100 MHz) data of **2** (in DMSO-*d*_6_).

No.	2				
	δ_C_, Mult	δ_H_, Mult, (*J* in Hz)	^1^H-^1^H COSY	HMBC	ROESY
1	21.9, CH_3_	2.41, br s	3, 15	2, 3, 15	
2	148.8, qC				
3	107.2, CH	6.88, br s	1, 15	1, 4, 13, 15	
4	155.2, qC				
5	168.8, qC				
6	136.5, qC				
7	111.8, CH	6.99, d, (2.4)	9	5, 8, 9, 11	8-OCH_3_
8	164.8, qC				
9	101.2, CH	7.20, d, (2.4)	7	7, 8, 10, 11	8-OCH_3_
10	157.6, qC				
11	109.7, qC				
12	179.2, qC				
13	105.9, qC				
14	160.5, qC				
15	111.2, CH	6.67, br s	1, 3	1, 3, 13, 14	
8-OCH_3_	56.6, CH_3_	3.96, s		8	7, 9
5-OH *		13.23, br s			
14-OH *		12.33, br s			

*: The assignment maybe exchanged.

## Supplementary Materials

Supplementary materials can be accessed at: http://www.mdpi.com/1420-3049/21/6/764/s1.
