# Supplementary material for: A New Xanthone Glycoside from the Endolichenic Fungus Sporormiella irregularis
Source: Molecules. 2016 Jun 11;21(6):764. doi: 10.3390/molecules21060764 (PMC6273424; doi:10.3390/molecules21060764)
Supplement: Supplementary file 1 [file molecules-21-00764-s001.pdf]

Supplementary Materials: A New Xanthone Glycoside from the Endolichenic Fungus *Sporormiella irregularis*

Bin-Jie Yang, Guo-Dong Chen, Yan-Jun Li, Dan Hu, Liang-Dong Guo, Ping Xiong, and Hao Gao

The 1D and 2D NMR spectra of 1 and 2

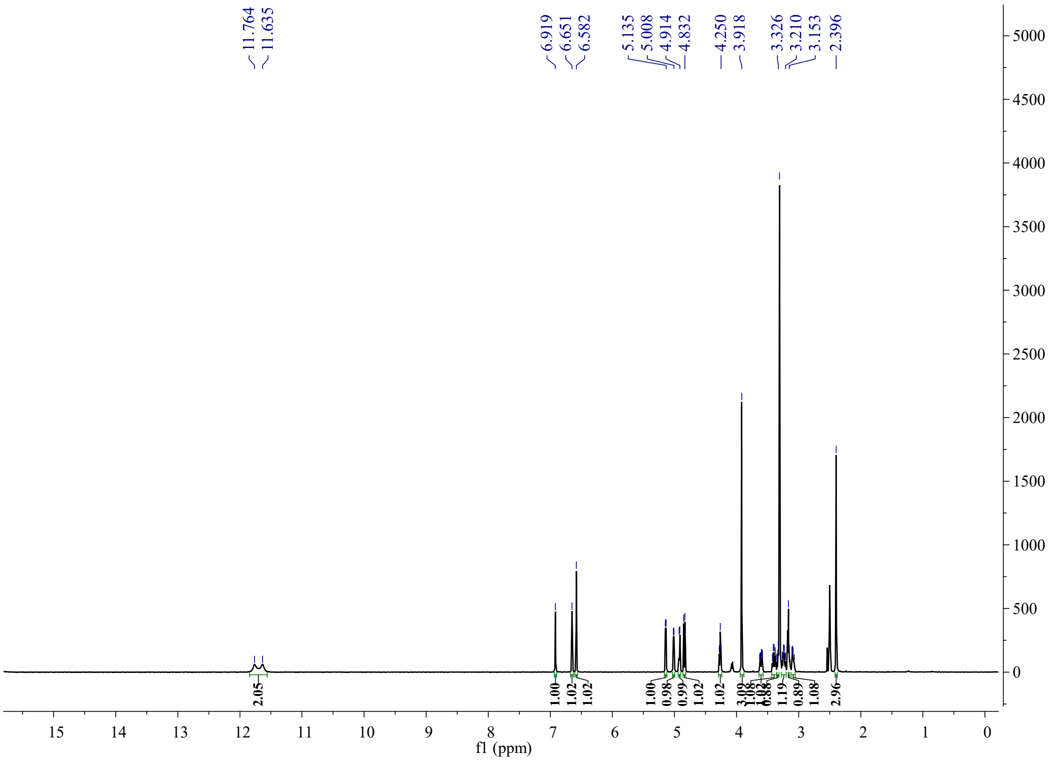

Figure S1. ^{1}H-NMR spectrum of **1** (400 MHz, in DMSO-d_6).

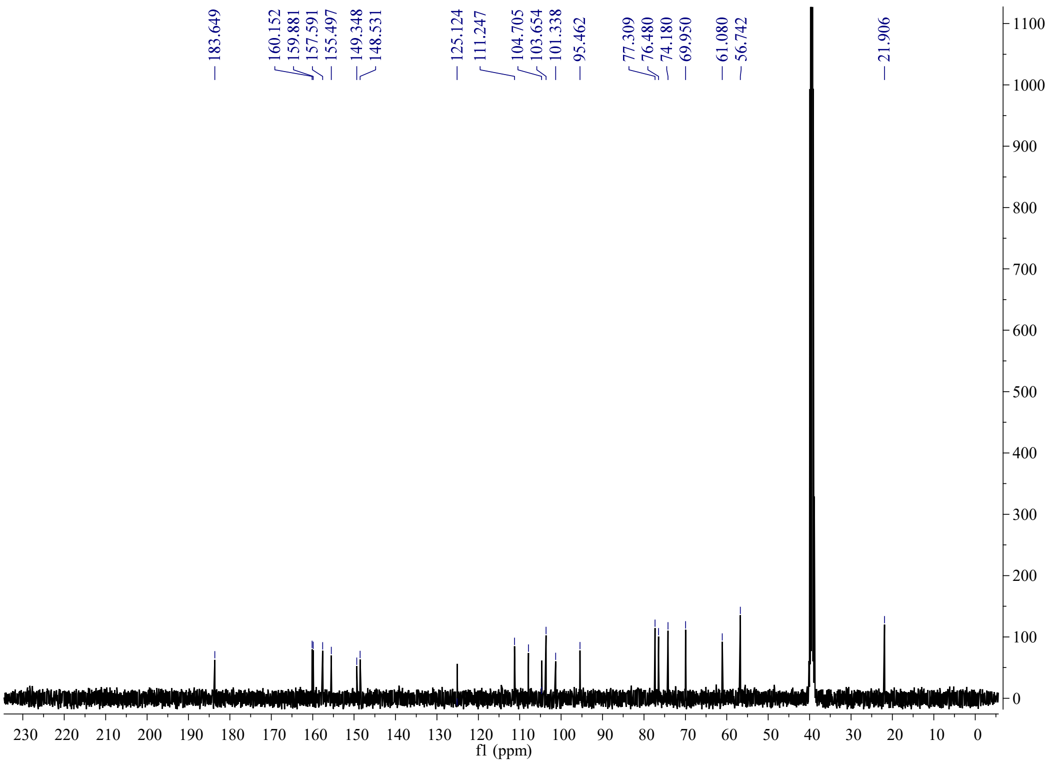

Figure S2. ^{13}C-NMR spectrum of **1** (100 MHz, in DMSO-d_6).

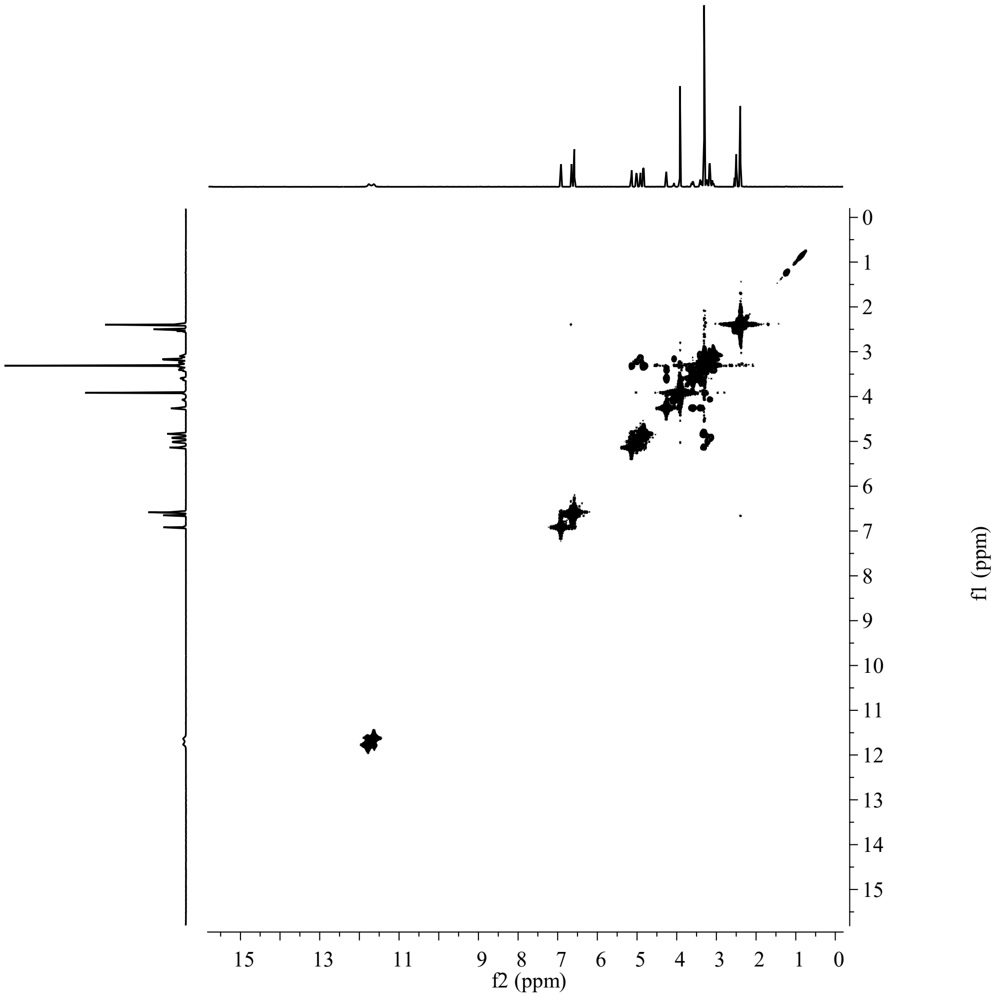

Figure S3. ^{1}H-^{1}H COSY spectrum for **1** in DMSO-d_6.

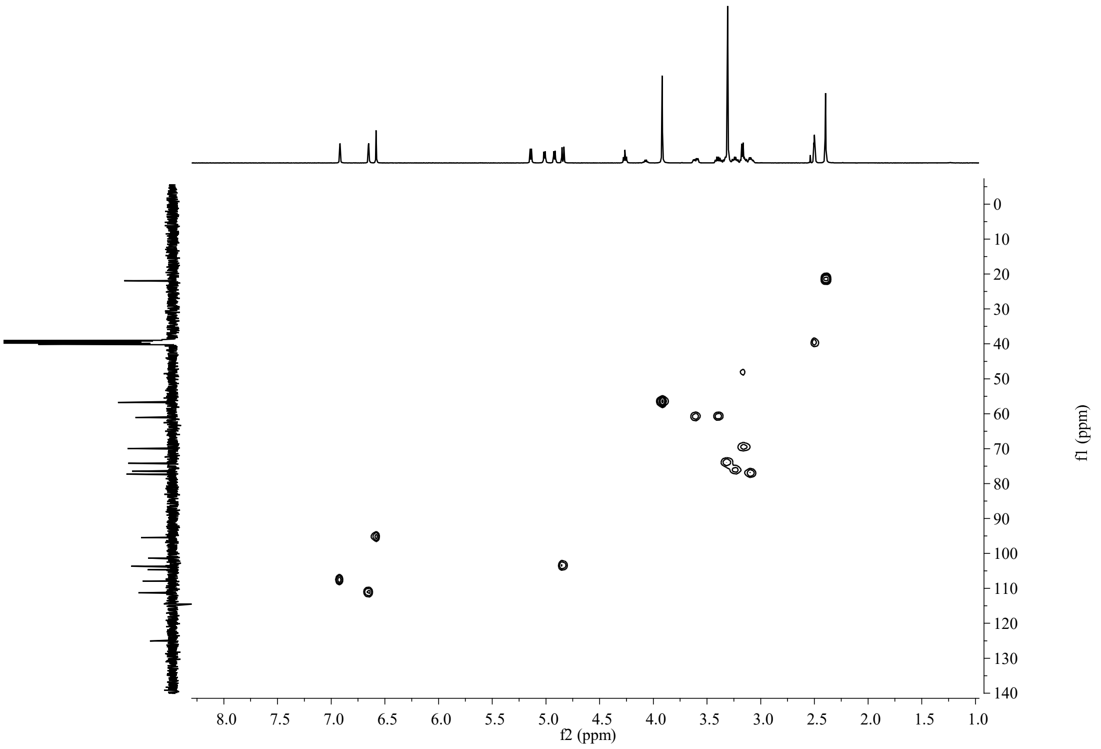

Figure S4. HSQC spectrum for **1** in DMSO-*d*6.

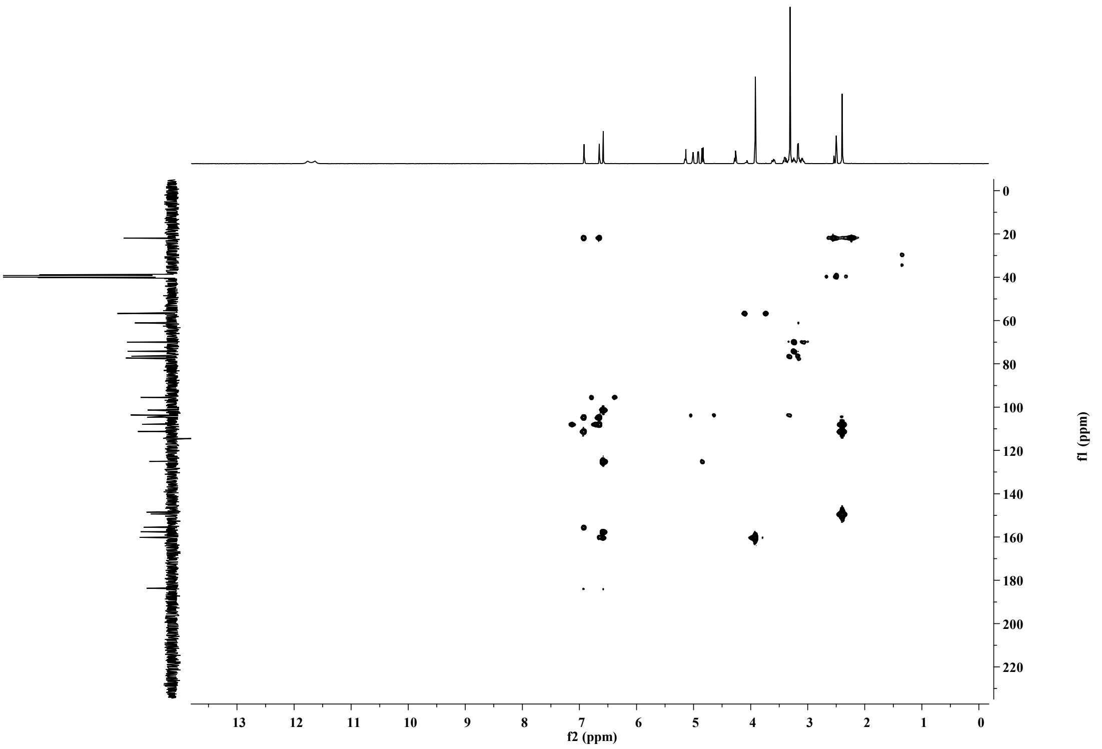

Figure S5. HMBC spectrum for **1** in DMSO-*d*6.

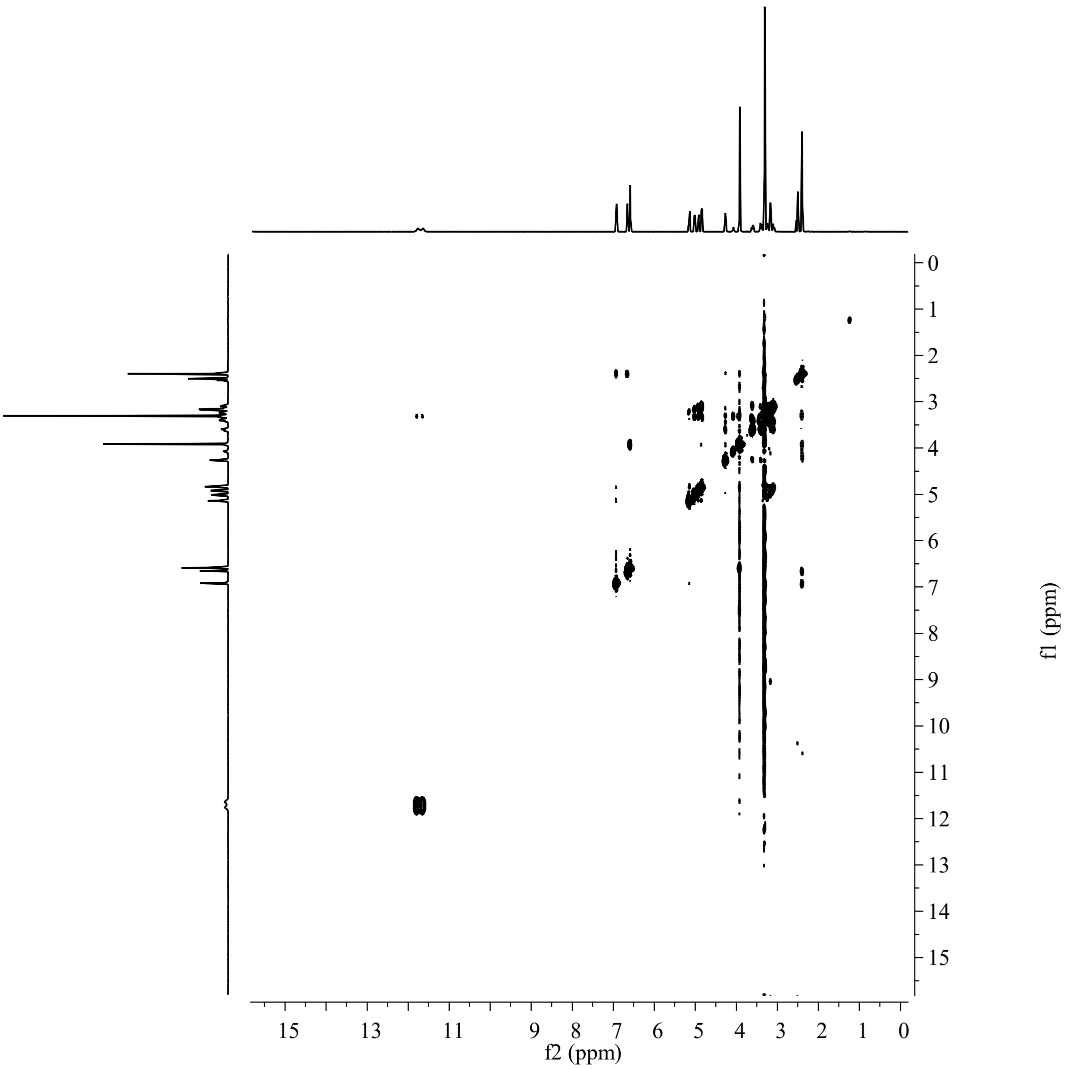

Figure S6. ROESY spectrum for **1** in DMSO-d_6.

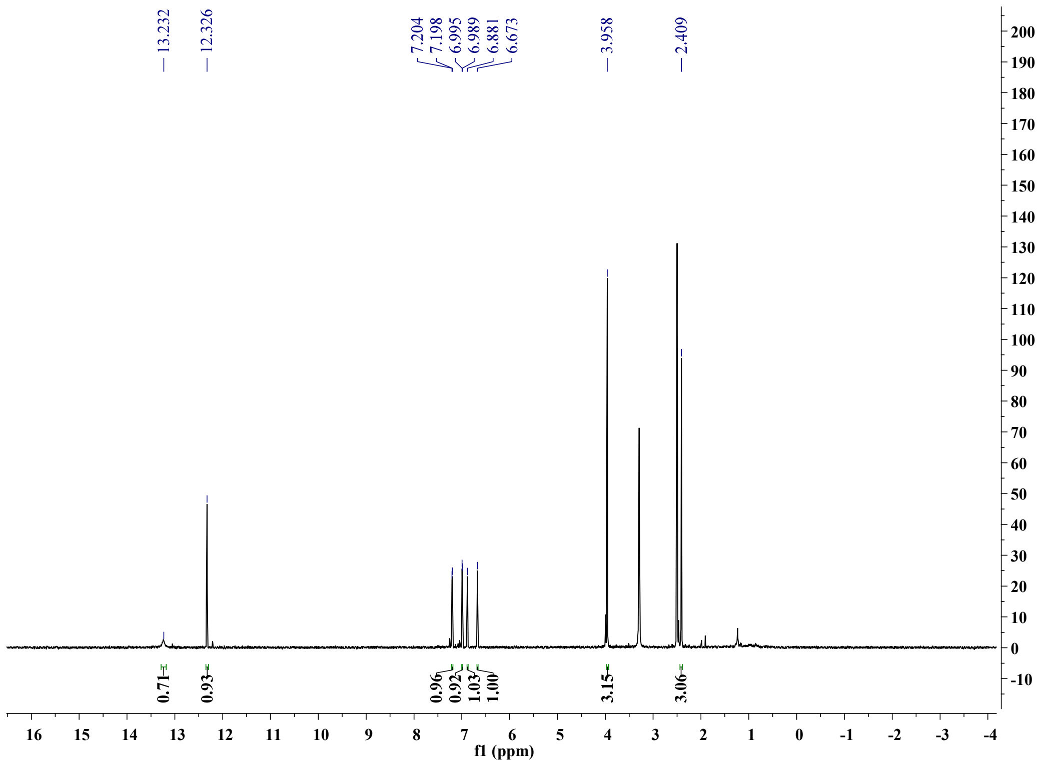

Figure S7. ^{1}H-NMR spectrum of **2** (400 MHz, in DMSO-d_6).

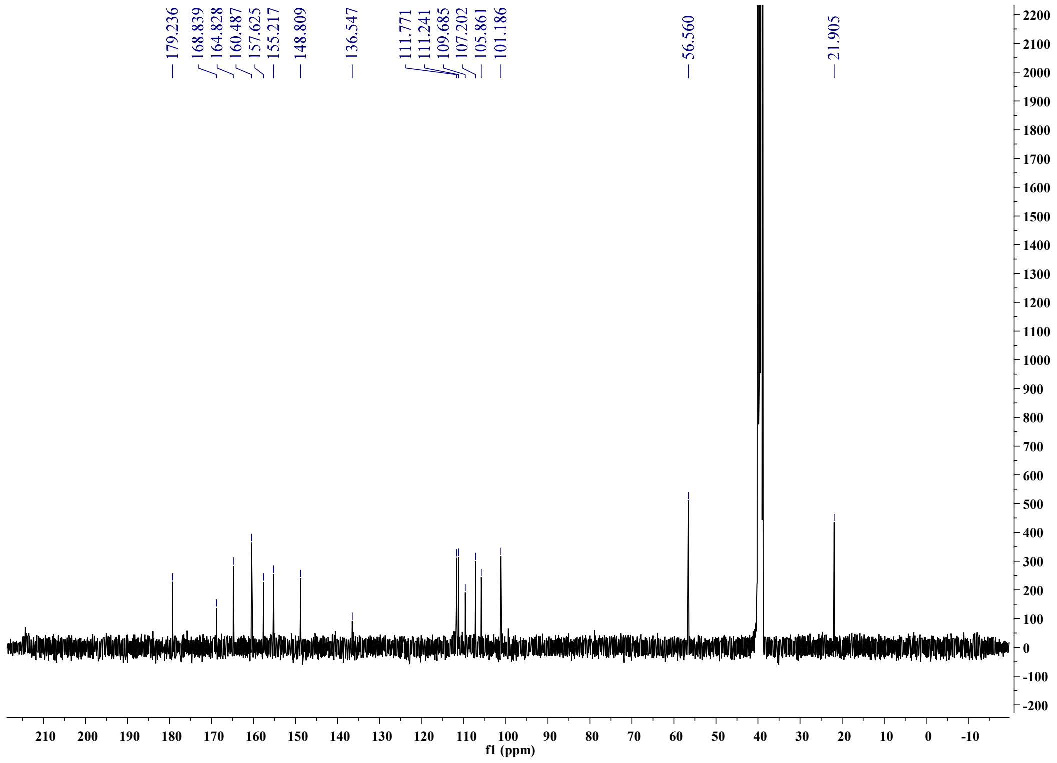

Figure S8. ^{13}C-NMR spectrum of **2** (100 MHz, in DMSO-d_6).

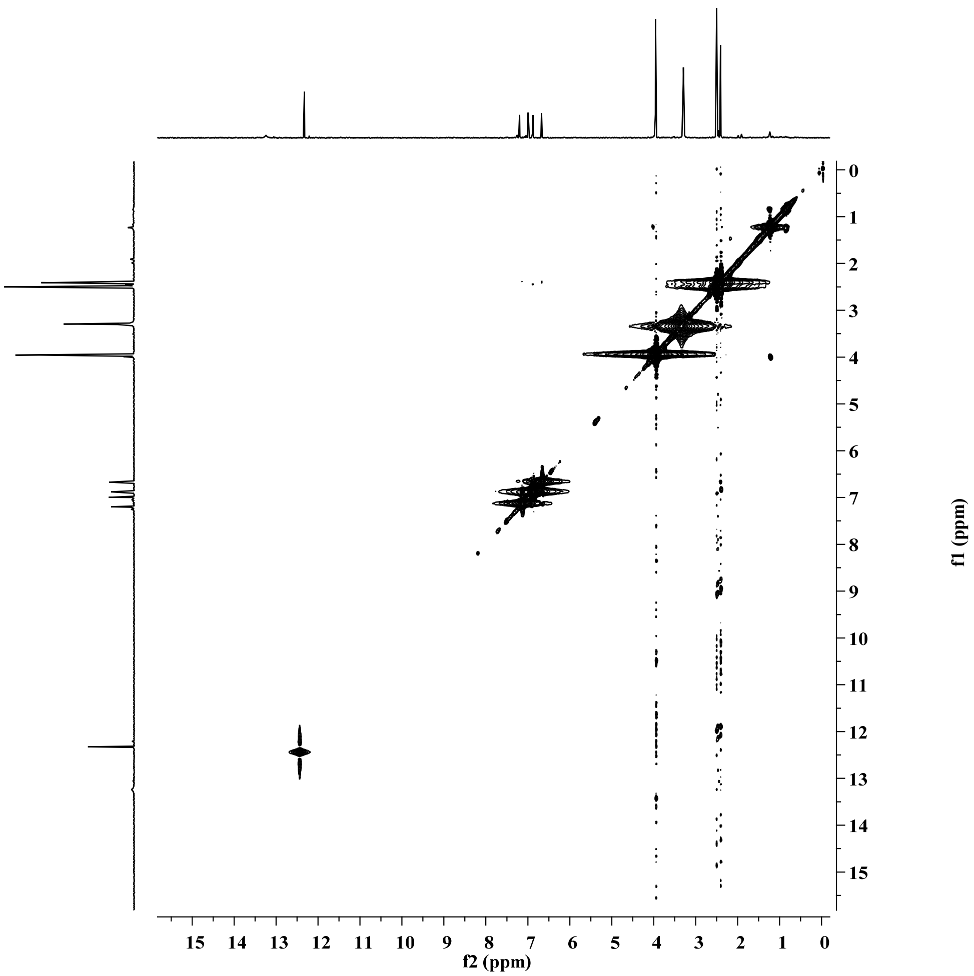

Figure S9. ^1H-^1H COSY spectrum for **2** in DMSO-*d*$_6$.

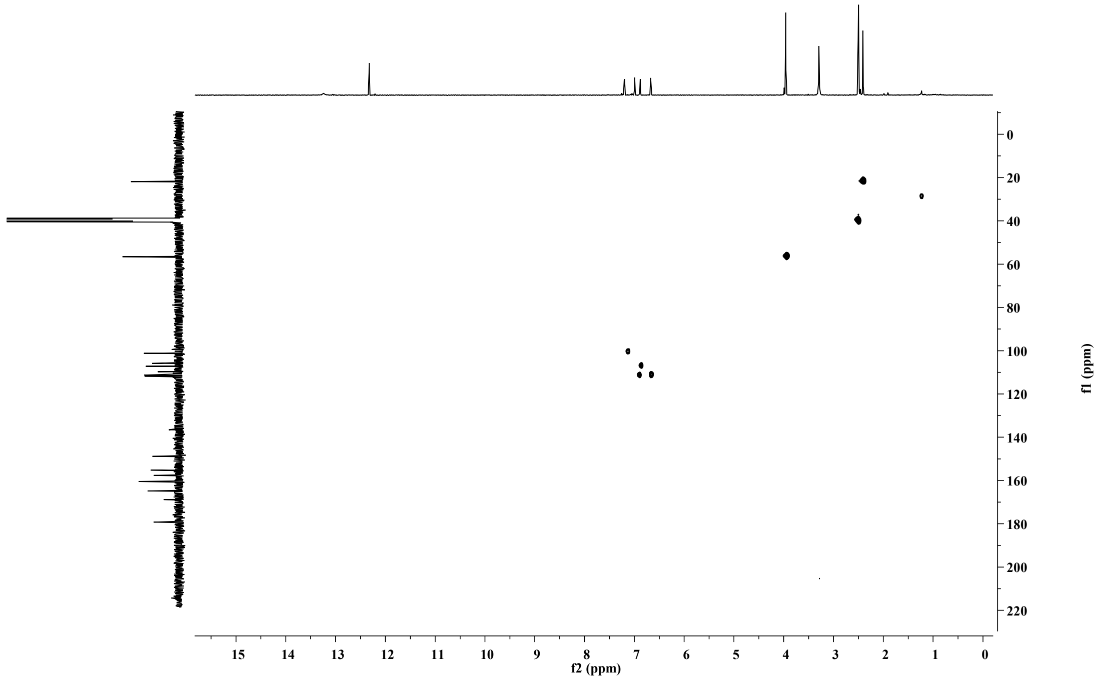

Figure S10. HSQC spectrum for **2** in DMSO-*d*$_6$.

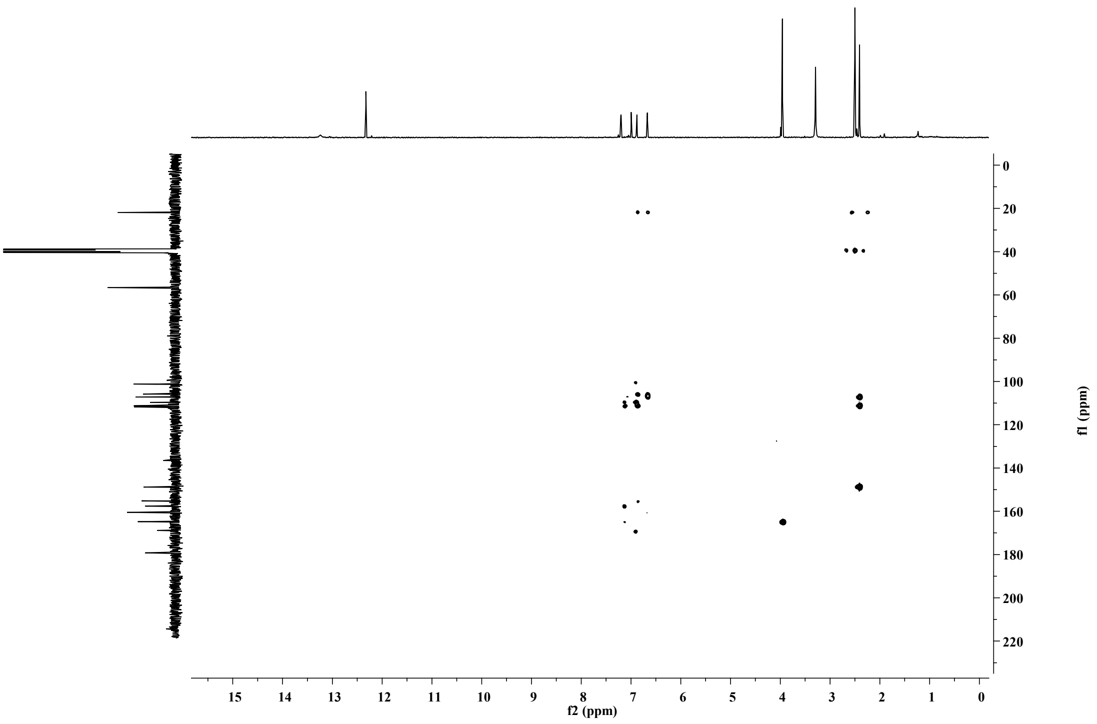

Figure S11. HMBC spectrum for **2** in DMSO-d_6.

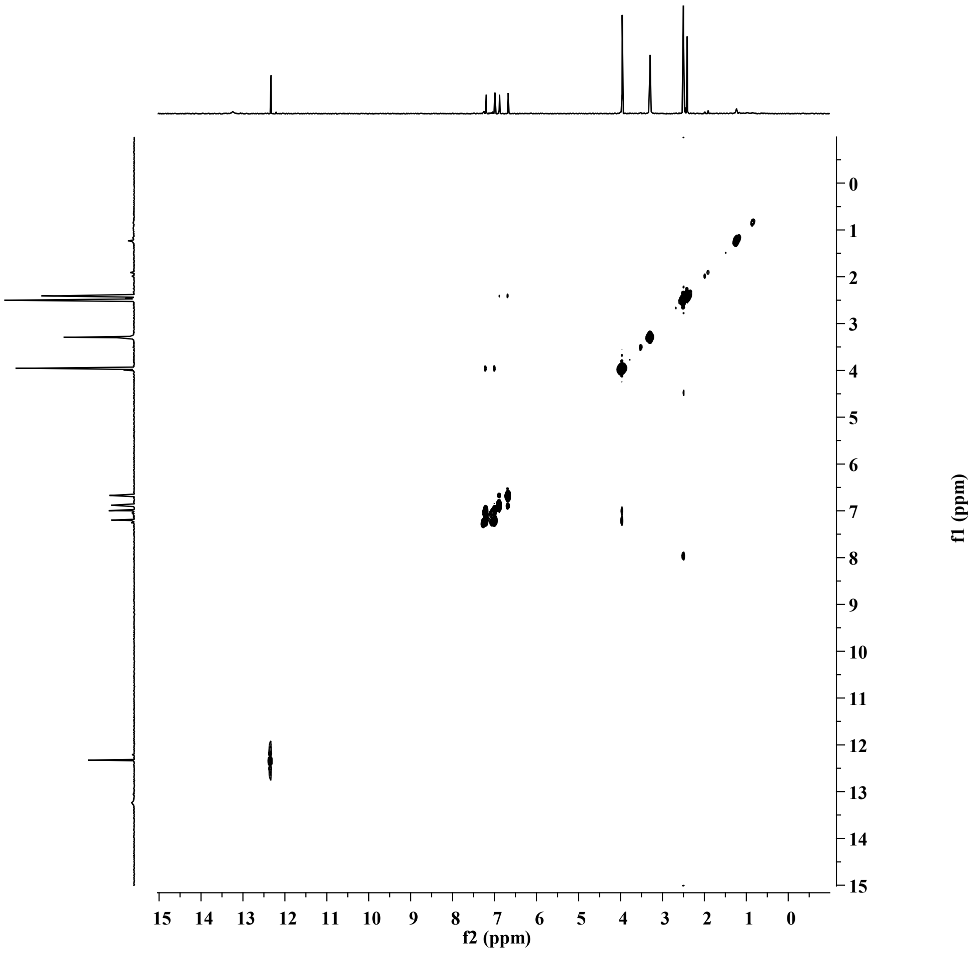

Figure S12. ROESY spectrum for **2** in DMSO-d_6.
